# Monomeric Amyloid Beta Peptide in Hexafluoroisopropanol Detected by Small Angle Neutron Scattering

**DOI:** 10.1371/journal.pone.0150267

**Published:** 2016-02-26

**Authors:** Bo Zhang-Haagen, Ralf Biehl, Luitgard Nagel-Steger, Aurel Radulescu, Dieter Richter, Dieter Willbold

**Affiliations:** 1 Jülich Centre for Neutron Science & Institute of Complex Systems, Neutron Scattering (JCNS-1&ICS-1), Research Centre Jülich, Jülich, Germany; 2 Institut für Physikalische Biologie, Heinrich-Heine-Universität Düsseldorf, Düsseldorf, Germany; 3 Institute of Complex Systems, Structural Biochemistry (ICS-6), Research Centre Jülich, Jülich, Germany; 4 Jülich Centre for Neutron Science, Outstation at MLZ (JCNS-MLZ), Research Centre Jülich, Garching, Germany; University of Akron, UNITED STATES

## Abstract

Small proteins like amyloid beta (Aβ) monomers are related to neurodegenerative disorders by aggregation to insoluble fibrils. Small angle neutron scattering (SANS) is a nondestructive method to observe the aggregation process in solution. We show that SANS is able to resolve monomers of small molecular weight like Aβ for aggregation studies. We examine Aβ monomers after prolonged storing in d-hexafluoroisopropanol (dHFIP) by using SANS and dynamic light scattering (DLS). We determined the radius of gyration from SANS as 1.0±0.1 nm for Aβ_1–40_ and 1.6±0.1 nm for Aβ_1–42_ in agreement with 3D NMR structures in similar solvents suggesting a solvent surface layer with 5% increased density. After initial dissolution in dHFIP Aβ aggregates sediment with a major component of pure monomers showing a hydrodynamic radius of 1.8±0.3 nm for Aβ_1–40_ and 3.2±0.4 nm for Aβ_1–42_ including a surface layer of dHFIP solvent molecules.

## Introduction

A common pathologic hallmark of neurodegenerative disorders like Alzheimer’s, Parkinson’s or Huntington’s disease is the existence of amyloid deposits in the brain[1–6]. Amyloid is generated by abnormal protein aggregation leading to formation of insoluble protein fibrils with a highly ordered cross-beta sheet structure. At the beginning of the 20th century beta amyloid protein (Aβ) was supposed to be associated with Alzheimer disease (AD) in consequence of the detection of Aβ fibril in intercellular plaques found in brain of AD patients[7]. Aβ fibrils and soluble oligomers are suggested to be responsible for the disease symptoms, whereas the monomers in the brain of AD patients are considered as nontoxic[8–10]. Aβ is a hydrophobic protein with 39–43 amino acid residues, which is produced by proteolytic cleavage of amyloid precursor protein (APP) associated with the cell membrane[11]. The γ-secretase, within the membrane region, generates the fibrillogenic C-terminus of Aβ[12] and may cause an amount of isoforms dependent on the exact position for cleavage, where Aβ_40_ and Aβ_42_ are the most common, while the extracellular β-secretase cleaves N-terminally. The residues Leu17-Ala21 constitute the hydrophobic core of Aβ. The residues Lys28-Ala42 and Gly9-Ala21 are capable to form α-helical or β-sheet structure, where the *β*-sheet is the priority for Lys28-Ala42[13,14]. As the key of AD investigation Aβ monomers have been studied by different methods as nuclear magnetic resonance (NMR), analytical ultracentrifugation (AUC), circular dichroism (CD) and Fourier transform infrared spectroscopy (FTIR)[15–18]. In aqueous environment the monomeric Aβ is mainly unstructured[19], whereas apolar solvents induce a folded, α-helical structure[20,21]. The conformational change to β-sheet is supposed as the first step of the aggregation process and has been observed by CD and NMR spectroscopy by increasing the content of water in apolar solvents (hexafluoroisopropanol (HFIP), trifluoroethanol (TFE))[17,22,23]. The classic hypothesis of fibril formation suggests monomer addition to soluble oligomers or to the seeds for fibril formation, whereas current explorations propose firstly the assembly of oligomers and secondly the structural conversion to β-sheet structure as alternative[16]. A nucleation like mechanism was used to explain the fibrillation in former studies[24].

The fast conformational change with rapid onset of the aggregation process makes a study of the early stage of aggregation from monomers to small oligomers and fibril formation in aqueous solvents challenging. High-resolution structural methods fail to deliver the needed information to understand disease mechanism, therefore low-resolution methods are important to gain insight in structure and kinetics of the aggregation processes. Non-destructive methods like dynamic light scattering (DLS) either give limited amount of information (only hydrodynamic radius) or, like small angle X-ray scattering (SAXS) techniques, may have a strong influence on the aggregation due to radiation damage associated with high intensity X-ray sources. Methods like atomic force microscopy, analytical ultracentrifugation or transmission electron microscopy on the other hand give a snapshot of the aggregation process.

For all of the mentioned methods the preparation of the initial sample of Aβ is crucial as it defines the starting point for examination of aggregation pathways. Different methods involving alkaline or acidic solutions or organic solvents are regularly used[25–28]. Solvents with rich fluoride content as HFIP or TFE are known to produce stable amyloid protein monomer solution and may lead to monomer dissociation from protofibrils/fibrils or break up of preaggregates[28–30]. After re-purification and before the addition of aqueous buffers the proteins will be dried and consequently the protein concentration is much higher than in experiment before the complete evaporation of HFIP.

Neutron scattering, in contrast to X-ray scattering, shows no radiation damage or radiation induced aggregation even on delicate samples like unfolded proteins[31]. Small angle neutron scattering (SANS) accesses the same low-resolution structural information as SAXS with a different scattering contrast as X-rays interact mainly with the electrons of the atoms and neutrons interact with the nucleus[32,33]. The scattered intensity depends on the isotopes allowing contrast variation by isotope exchange[34] as e.g. using D_2_O instead of H_2_O to reduce the scattering of the solvent. SAXS and SANS are frequently used to determine the low-resolution structure of proteins and protein complexes in solution[32,35]. By inelastic neutron scattering methods the internal dynamics between domains on several nanosecond timescale or the sidechain motions on picosecond timescales can be observed[36,37].

So far, SANS was rarely used to study aggregates and aggregation kinetics of small peptides like Aβ or insulin, despite the advantage that it might reveal new structural information that is not accessible by other methods and in contrast to SAXS does not induce radiation damage in the sample[38–42]. Typically small angle scattering is used to determine the size or shape of larger aggregates during aggregation. Discrimination between populations of monomers, small oligomers and fibrils is challenging because of the low concentrations used in aggregation studies below 1 mg/ml and limited solubility of the peptides. Additionally discrimination is only possible if clear differences between populations can be expected as in case of Aβ where monomers, oligomers and large fibrils are present[43,44].

To examine the possibilities and limitations of small angle scattering techniques to observe the monomer to oligomer transition exploiting the nondestructive character of neutrons as a probe for delicate proteins, the monomer scattering is examined here as a limiting case for further aggregation studies. In biological relevant environment (H2O or D2O buffers) the Aβ monomer scattering is low and hardly to detect at lower concentrations as it is close to the SANS instrument noise and the fast aggregation introduces aggregates/oligomers masking a clear signal of the present monomers. Therefore we examined a monomeric solution in dHFIP to show what can be expected from the scattering signal and which effects need to be included in interpretation. This will help in the evaluation of aggregation studies by SANS to discriminate between monomers and oligomers. We examine here monomeric solutions of Aβ_1–42_ and Aβ_1–40_ as the most common peptides for Aβ aggregation studies at relative high concentration after incubation in dHFIP for several days. We observe a stable monomer population and a disappearing aggregate population by DLS and examine the monomeric solution by SANS. We demonstrate that SANS is capable to resolve monomeric Aβ with different radius of gyration for Aβ_1–42_ and Aβ_1–40_ and strong indications for a solvent surface layer bound to the peptides.

## Materials and Method

### Materials

The Aβ peptide was purchased as TFA salt with >95% purity (Bachem, Weil am Rhein, Germany). The deuterated 1,1,1,3,3,3-hexafluoro-2-propanol-D2 (dHFIP) was purchased from euriso-top (Saint-Aubin, France) with >99% purity. Aβ was dissolved in dHFIP in the purchased vials to yield a concentration of about 12 mg/ml dry mass including remaining salt or interfacial water from the protein powder. The peptide concentration was determined by UV absorption measurement (NanoDrop, Thermo Scientific, USA), applying an extinction coefficient at 280 nm of 1490 cm^-1^M^-1^, based on the single tyrosine residue of Aβ[45]. Final protein concentrations were about 5.6 mg/ml for Aβ_1–42_ and about 2.4 mg/ml for Aβ_1–40_. No further purification was done to prevent any additional decrease of concentration or material loss. Incubation and all measurements for Aβ_1–40_ and Aβ_1–42_ were performed at 20°C. SANS experiments were carried out after three weeks incubation in dHFIP.

### Small Angle Neutron Scattering (SANS)

Experiments were performed at the high intensity SANS diffractometer KWS2 operated by Jülich Center for Neutron Science at Heinz Maier Leibnitz Zentrum (MLZ) in Germany at a wavelength *λ* = 4.7 Å with a wavelength spread of *λ/Δλ* = 0.2 [46]. The samples were measured in 1 mm quartz cells (Hellma Analytics, Germany) at sample-detector-distances 1.1 m and 5.6 m covering the range of scattering vector *Q* = 4*π/λsin*(*θ*/2), with scattering angle θ, between 0.01 Å^-1^ and 0.5 Å^-1^. Samples and buffer measurements for background correction were measured for 1 h to 3 h dependent on detector distance. Appropriate standard methods for data evaluation and background correction were used.

The coherent scattering intensity of particles in solution with background contributions *bgr* is

$$I\left(Q\right)\;=\frac{N}{V}S\left(Q\right)F\left(Q\right)+bgr$$ (1)

with the number of particles *N* in the volume *V*. Background *bgr* contributions are due to scattering from solvent and incoherent particle scattering. The interparticle interactions are subsumed in the structure factor *S*(*Q*), which is for low concentrations equal one and is neglected in the following. The scattering of the protein configuration is described by the form factor *F*(*Q*).

$$F\left(Q\right)\;=\sum_{i.j}\langle b_{i}b_{j}exp\;\left(iQ\left(r_{i}-r_{j}\right)\right)\rangle$$ (2)

with the atomic position vectors *r*_*i*_ and the atomic scattering lengths *b*_*i*_ relative to the displaced solvent scattering in the ensemble average <^.^>[47]. The form factor is independent of the sample concentration and exhibits the particle shape, respectively the configuration. Additional scattering from a surface layer of solvent around the protein can be taken into account by assuming a layer of solvent with increased density compared to the bulk[47].

### Models

The Beaucage function is a simple and common model being applied to analyze SANS data of objects with undefined geometry. It describes the Guinier region at low *Q* with radius of gyration *R*_*g*_ as a measure of size and a power-law at high *Q* with dimensionality *d* as measure of shape or configuration[48–50] as

$$\begin{array}{c} F\left(Q\right)\;=Gexp\;\left(-\frac{Q^{2}R_{g}^{2}}{3}\right)+\left(\frac{C}{Q^{d}}\right)\;{\left[erf\;\left(\frac{QR_{g}}{\sqrt{6}}\right)\right]}^{3d}\\ C\;=\left(\frac{Gd}{R_{g}^{d}}\right)\;{\left[\frac{6d^{2}}{\left(2+d\right)\left(2+2d\right)}\right]}^{d/2}Г\left(\frac{d}{2}\right) \end{array}$$ (3)

with the Guinier scaling factor *G*, the Porod scaling factor *C*, error function *erf* and Gamma function *Γ*. With *d* = 2 it is a good approximate for the Gaussian coil[50].

Atomic modeling is based on the atom positions of PDB structures and Eq 2 taking into account the scattering length difference relative to the solvent. To consider the excluded solvent scattering the solvent excluded volume V_SES_ needs to be determined. Therefore the protein solvent accessible surface (SAS) is calculated with a probe radius R_p_ = 0.14 nm resulting in volume V_SAS_ and surface area A_SAS_ as implemented in the used MMTK software[51,52]. As a fast approximate for V_SES_ we subtract the surface layer volume with a correction for overlapping regions in grooves determined from the additional surface area with half the probe size as V_SES_ = V_SAS_-R_p_×(A_SAS_(R_p_)+0.5(A_SAS_(R_p_)-A_SAS_(R_p_/2)). The resulting specific volumes are 0.705 cm^3^/g and 0.720 cm^3^/g for Aβ_1–40_ and Aβ_1–42_ as average over the configurations in pdb structures 1IYT and 1AML, respectively. Both values are slightly smaller compared to reported values from analytical ultracentrifugation of 0.734 cm^3^/g and 0.738 cm^3^/g for Aβ_1–40_ and Aβ_1–42_[18,53]. With these increased values for the specific volume the resulting scattering would increase.

### Dynamic Light Scattering (DLS)

Experiments were performed with a Zetasizer Nano ZS (Malvern Instruments, Worcestershire, UK). The instrument uses a He-Ne laser with *λ* = 632.8 nm and vertical polarization in backscattering geometry at 173°. The sample cell is a UV-Cuvette micro (BRAND, Wertheim, Germany) with 70 μl sample volume. To cover also larger aggregates (as the later third species) the measurements duration was set to 1h to get reliable data at long times. The auto correlation function was analyzed by non-negative least square (NNLS) algorithm[54] followed by protein analysis (*L*-curve)[55] as implemented in the instrument software. The result is an intensity weighted distribution of hydrodynamic radius *R*_*H*_ in the sense of a relaxation time distribution where each relaxation time *τ* is interpreted via the Stoke-Einstein relation *R*_*H*_ = *k*_*B*_*T/*6*πηD* with the diffusion coefficient *D* = (*Q*^*2*^*τ*)^-1^ and *Q* as the scattering wave vector, Boltzmann constant *k*_*B*_, temperature *T* and solution viscosity *η*. The mean *R*_*H*_ and the width of the resulting distribution characterize a population. It should be noted that the typical noise of the measured auto correlation results in a distribution width of about 30% for an ideal monodisperse population. The error of a mean *R*_*H*_ is evaluated as standard deviation of repeated measurements. The volume fraction of a population can be evaluated from the volume-weighted distribution as calculated by the instrument software.

## Results and Discussion

**Fig 1:**
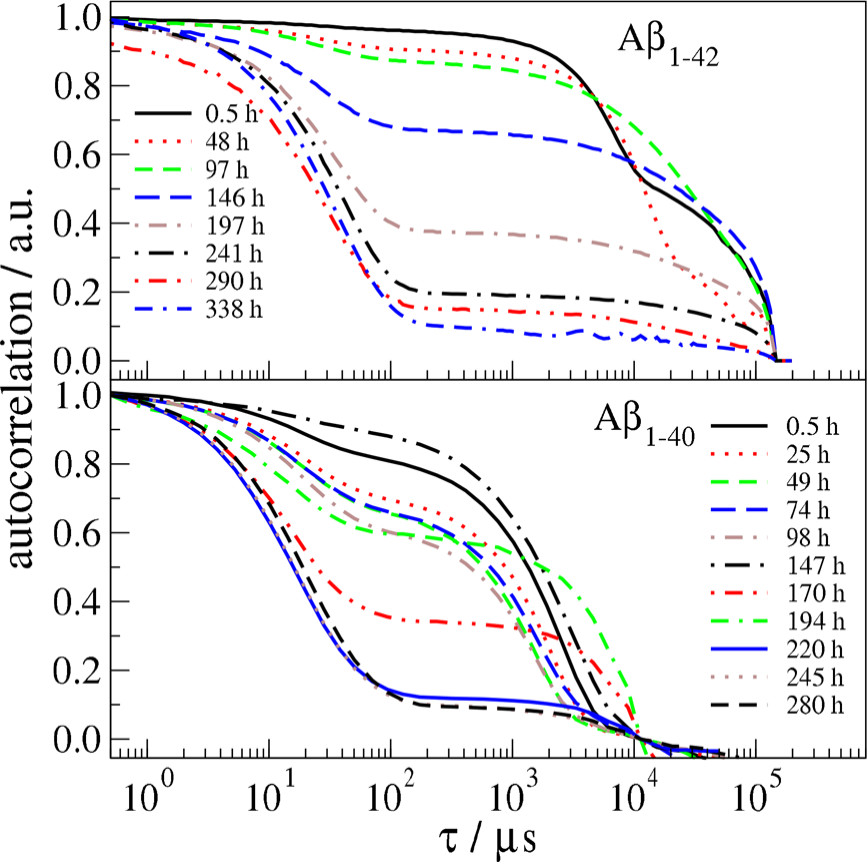
Time resolved DLS experiment of Aβ_1–42_ and Aβ_1–40_ in dHFIP. The samples were measured between 0.5 h and 14 days after initial incubation at 20°C. For Aβ_1–40_ the value of *τ* = 10000 μs is subtracted to focus on the two smaller species. For both samples correlations are normalized to 1 for ***τ*→0** to get a better overview over the data. Original measurements are found in S1 and S2 Figs.

To examine the content of freshly in dHFIP dissolved Aβ powder we explored the evolution of the intensity correlation of Aβ_1–40_ and Aβ_1–42_ in dHFIP at 20°C by repeated DLS experiment. Shortly after dissolution three distinct particle populations can be observed (see Figs 1 and S1). The population with largest *R*_*H*_ belongs to peptide aggregates or fibrils (or contaminating dust particles) with sizes larger than several microns. The remaining two populations show mean relaxation times of about 20 μs and 5000 μs corresponding to hydrodynamic radii *R*_*H*_ around 2 nm and 500 nm, which are clearly separated in the auto correlation function as shown in Fig 1. The population with *R*_*H*_ ≈ 2 nm and width typical for monomeric protein solutions refers to monomers. Analysis of the volume weighted distributions shows that the monomer population is the main component to the peptide content with more than 99% for all measurements. Accordingly the concentration as measured by UV-Vis absorption does not change significantly with time. The two larger populations refer to aggregates that strongly contribute to the scattering intensity because the scattered intensity is proportional to the sixth power of the particle size. During the observation time the size of the second population varied between 100 nm and 3000 nm, where the content fluctuated, but mainly shows a decrease of the scattering contribution. A change in size may be caused by monomer dissociation/aggregation from aggregates, fragmentation/aggregation of aggregates or faster sedimentation of larger aggregates. For the cases of dissociation, aggregation and fragmentation we expect an obvious change of size including sizes smaller than 100 nm (relaxation times <1000 μs), which is not observed in detectable amount. Therefore, we supposed that the dominating process for both larger aggregate species is sedimentation during the incubation in dHFIP. Changes of the aggregate contribution to the auto correlation may result from wobbling of the sample prior to the measurement. After about 9 days incubation in dHFIP there is no more larger change in the correlation function observable. The sample reached its equilibrium and contains hardly any larger aggregates. The small particles have a hydrodynamic radius of about 1.8±0.3 nm for Aß_1-40_ and 3.2±0.4 nm for Aß_1-42_.

**Fig 2:**
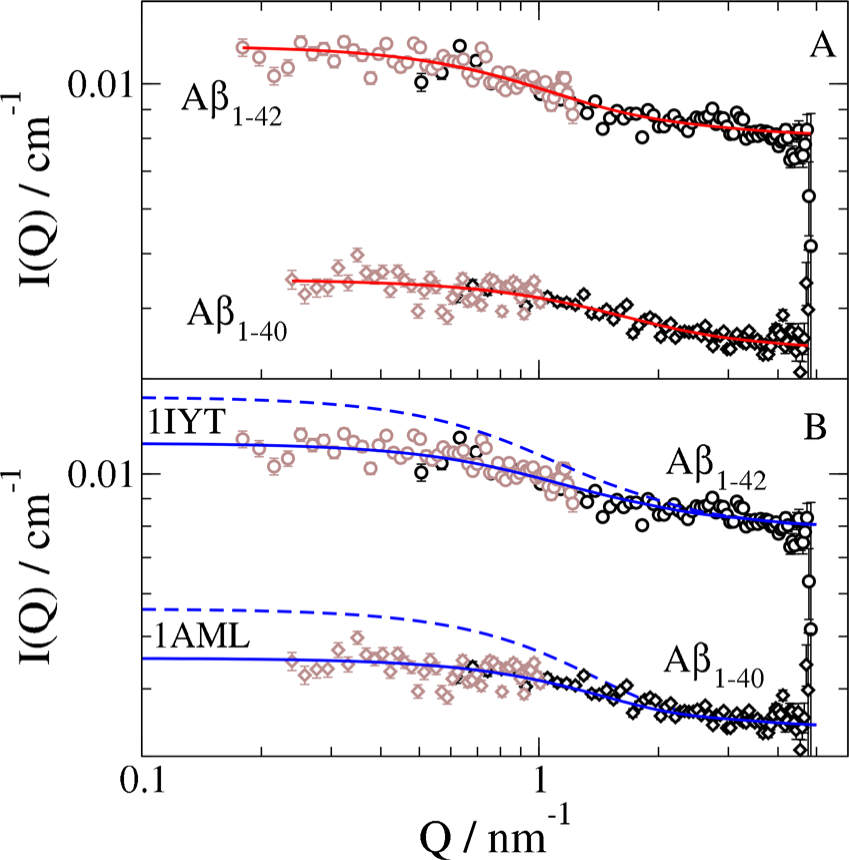
Scattering intensity of Aβ monomers in dHFIP (Aß_1-42_, concentration 5.6 mg/ml, Aß_1-40_ concentration 2.4 mg/ml) after three weeks incubation measured at detector distance of 5.6 m (grey) and 1.1 m (black). (A) The red solid line shows the fitted Beaucage function with fixed dimensionality ***d* = 2**. (B) SANS data compared to simulations based on atomic coordinates from 3D NMR structure data (Aß_1-42_ PDB code 1IYT, Aβ_1–40_ compared to PDB code 1AML). Broken blue lines represent the average over the PDB structures; while solid blue lines include a surface layer with increased solvent density.

Fig 2 shows the SANS scattering curves of Aβ in dHFIP. In spite of the relatively high concentrations—compared to aggregation studies with concentrations below 1 mg/ml—the intensity is quite low due to the small size of Aβ and about a factor of 3 below the background of pure dHFIP that is subtracted taking the protein volume fraction into account. A residual background at high *Q* is obvious, which is due to incoherent scattering from the protein (about 0.0025 cm^-1^ for the 5.6 mg/ml) and residual scattering from protonated water and salts in the purchased protein powder. The difference in forward scattering of about a factor of 3 corresponds mainly to the difference in concentration. Low intensity and residual background favour together a Beaucage model including a background over the classical Guinier analysis for Rg evaluation as the later does not include a background contribution[56]. The measured intensity was modeled by a Beaucage function with fixed dimensionality (*d* = 2) within a least square fit including the residual background. The radius of gyration *R*_*g*_ evaluates to 1.6±0.1 nm and 1.0±0.1 nm for Aβ_1–42_ and Aβ_1–40_ respectively (see Fig 2A).

**Fig 3:**
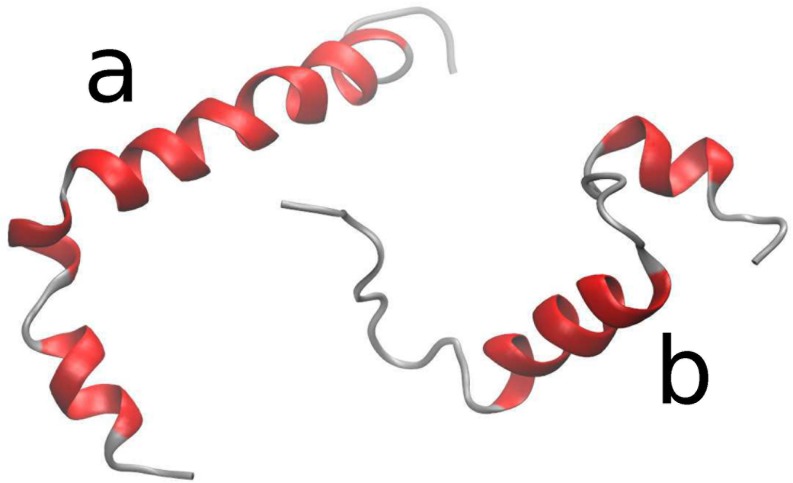
Exemplary structure of Aβ monomers: (a) Aβ_1–42_ dHFIP_0.8_D_2_O_0.2_ (PDB code 1IYT)[20] with two α helices from residues Ser8-Gly25 and Lys28-Gly38, which is connected by a type I β-turn. (b) Aβ_1–40_ in TFE_0.4_H_2_O_0.6_ (PDB code 1AML)[21] with two α helices from Gln15-Asp23 and from Ile31-Met35 connected with a type I β-turn.

The 3D structure of Aß_1-42_ monomer in dHFIP_0.8_D_2_O_0.2_ is available from the PDB data bank (PDB code 1IYT) as measured by solution NMR (see Fig 3A) with a set of 10 conformations, which show mainly reorientations due to a small hinge between the two α-helices[20]. For Aβ_1–40_ a structure file (PDB code 1AML) measured in 40%TFE/water with 20 different conformations is available (see Fig 3B)[21]. The simulated scattering curves of the conformations are illustrated in S3 Fig and the averages are shown in Fig 2B as broken line for both structures. In both cases we find too strong scattering. Including the effect of a solvent surface layer with higher density compared to the bulk solvent comparable to the hydration layer observed for proteins in water[47] the calculated scattered intensity matches the measured intensity with an increase in density of 5% for a 0.3 nm thick solvent surface layer. The surface layer in water seems to be mainly determined by geometric effects of the surface and partly by the electrostatic field at the protein surface and water structure perturbation as shown by MD simulations by Merzel and Smith[57]. At least a similar geometric effect of the protein surface might be present here. MD simulations can only answer how far electrostatic effects or the stronger hydrogen bonds of HFIP compared to water contribute. The mean *R*_*g*_ of Aβ_1–42_ monomer from these conformations is 1.58 nm and 1.46 nm including the surface layer (see S3 Fig). For Aβ_1–40_ the mean *R*_*g*_ is 1.29 nm and 1.16 nm including the surface layer. Thus a surface layer with increased density decreases *R*_*g*_ as a result of the combination of solvent scattering length density and protein scattering length density. The strong effect of the surface layer is related to the large volume of the surface layer, which is in average a factor of 2.6 (1AML) and 2.3 (1IYT) larger than the protein volume. For a detailed determination of a changed density in the surface layer a combination of SAXS and SANS with different contrast contributions is required. SAXS measurements were not possible in dHFIP as the solvent evaporated due to the high-energy input on a high intensity beam line. Nevertheless the observed radius of gyration seems to be influenced by a solvent surface layer and shows compatibility of the observed radius of gyration with previously reported structures of Aβ in similar solvents. It should be noted that the actual configuration and flexibility has a strong influence on the scattering as SANS measures the configurational ensemble. Comparing the simulated scattering of the different configurations according to the PDB structures (see S3 Fig) we observe that the Aβ_1–42_ scattering patterns stay quite similar as the small hinge between the two α-helices allows only small changes of the configuration. Aβ_1–40_ has larger configurational freedom (more disordered regions and stronger variation in the PDB structures) and consequently larger differences in the scattering patterns and *R*_*g*_. Generally a larger configurational freedom may allow more extended configuration_._ Nevertheless, the NMR structures and the SANS data show a similar tendency with a more compact Aβ_1–40_ compared to Aβ_1–42_. The larger configurational freedom results here in a more compact structure, which may be a result of a reduced contact to the solvent.

The question arises how to compare the radius of gyration and the hydrodynamic radius as both are influenced by a solvent surface layer, but in different ways. A simple approach for globular proteins relates the radius *R*_*S*_ of a sphere to the radius of gyration by $R_{g}^{2}=\left(3/5\right)R_{s}^{2}$ resulting in *R*_*s*_ of 2.06 nm for Aβ_1–42_ and 1.3 nm for Aβ_1–40_. If we assume that the monomer is surrounded by a solvent layer contributing to the apparent hydrodynamic radius (2*R*_*s*,*HFIP*_ = 0.68 *nm*) the hydrodynamic radius of Aβ_1–42_ and Aβ_1–40_ monomer is about 2.7 nm and 2.0 nm respectively, which correspond approximately to the measured *R*_*H*_ from DLS experiment. In a different approach based on measurements of *R*_*g*_ and *R*_*H*_ for proteins between 50–400 residues in denaturing solvents the relation *R*_*g*_*/R*_*H*_ = 1.06 is found[58]. Even taking into account that Aβ is smaller than the reference proteins and the difference in solvent molecule size, too small hydrodynamic radii result demonstrating that a Aβ seems to be not in a disordered state.

The hydrodynamic radii of Aβ_1–42_ and Aβ_1–40_ in water is according to Nag et al. 0.9 nm[43]. For both monomers the actual conformation in water is not known and the influence of a hydration layer is undetermined. Assuming a hydration layer of 0.3 nm as found for other proteins the configuration seems to be rather compact. Nevertheless it is clear that not only the predominantly α-helical structure in dHFIP can increase the hydrodynamic radius as observed. The strong hydrogen bonding properties of dHFIP may be of importance here. Stronger hydrogen bonding compared to the peptide in water may cause a larger peptide-solvent complex that contributes to the hydrodynamic radius. For the smaller Aβ_1–40_ the more compact structure reduces the surface to the solvent and a lower number of solvent molecules may contribute to the complex resulting in the smaller hydrodynamic radius compared to Aβ_1–42_. The strong hydrogen bonding of dHFIP allows solvation of monomeric peptides without aggregation for Aβ and seems to increase the apparent hydrodynamic radius.

## Conclusions

In this study we investigated the applicability of SANS to the study of the Alzheimer’s disease associated amyloid beta peptide (Aβ). Several properties of this 4 to 5 kDa small peptide make structural studies with high-resolution methods of the monomer as well as of the early assembly states extremely difficult. Therefore, other methods giving less detailed structural information become more important. Small angle neutron scattering and dynamic light scattering at relatively high concentration (for Aβ aggregation experiments) was used to probe the application of SANS as a scattering method without radiation damage for Aβ solutions as a prerequisite for further kinetic measurements of Aβ aggregation and fibril formation. We found that the solvation of Aβ in dHFIP is a fast process, where after a short incubation of less than 1 h the remaining aggregates mainly sediment. Therefore preparation time can be shortened by centrifugation to sediment the small amount of aggregates. We showed that SANS is able to perform measurements with monomeric Aβ solutions to determine the radius of gyration. Although we cannot differentiate between conformations as in high-resolution techniques, we find a more compact structure of Aβ_1–40_ compared to Aβ_1–42_ showing that low resolution information can give valuable insight about the general structure. The difference in structure between Aβ_1–40_ and Aβ_1–42_ regarding the distribution of α-helices and disordered regions seems to be preserved in pure dHFIP. Additionally the results show the probable existence of a surface layer with increased density of dHFIP solvent similar to the hydration layer of proteins in water, which was not observed by other methods. The solvent surface layer increases the hydrodynamic radius considerably. Still the application of SANS onto low concentration samples of a protein as small as Aβ would benefit from higher intensity and it is at the limit of the nowadays possibilities.

## Supporting Information

S1 Dataset DLS and SANS data. (ZIP)

S1 Fig Time resolved DLS experiment with a concentration of 2.4 mg/ml Aβ_1–40_ in dHFIP. The sample was measured between 0.5 h and 12 days after initial incubation at 20°C. The strong change in the absolute value of the correlation function at ***τ* → 0** (intercept) is due to the increase in signal to noise ratio. With more large aggregates, respectively higher intensity, the signal has less noise and the intercept is closer to one. Additionally the signal to noise ratio depends on the laser attenuation chosen automatically by the instrument adjusting the mean intensity to values between 200000 and 500000 counts/s dependent on the total scattered intensity of a sample.
(EPS)

S2 Fig DLS of 5.6 mg/ml Aβ_1–42_ in HFIP. The sample was measured between 0.5 h and 14 days after initial incubation at 20°C in HFIP. See S1 Fig for more comments.
(EPS)

S3 Fig Simulated scattering intensities for configurations according to PDB structures of Aβ_1–42_ and Aβ_1–40_. Black and green lines take only the contrast to the dHFIP solvent into account, while for red and blue lines additionally a solvent surface layer with 0.3 nm thickness and 5% higher density compared to bulk dHFIP is used. The given radius of gyration ***R***_***g***_ is the average of the different structures in the PDB file as evaluated from the scattering in the Guinier region. The differences in the forward scattering for the same protein arise due to the changed protein volume if the configuration is changed.
(EPS)
